# Enhancing soybean (*Glycine max* L.) yield and quality through optimized weed-free periods and sowing techniques

**DOI:** 10.3389/fpls.2026.1700878

**Published:** 2026-02-16

**Authors:** Muhammad Awais Arshad, Rana Nadeem Abbas, Rania Baloch, Ali Ahmad, Usman Zulfiqar, Fasih Ullah Haider, Hossam S. El-Beltagi, Mashael Daghash Alqahtani, P. V. Vara Prasad

**Affiliations:** 1 Department of Agronomy, University of Agriculture, Faisalabad, Pakistan; 2 Department of Botany, Faculty of Sciences, University of Agriculture, Faisalabad, Pakistan; 3 Arid Zone Research Institute (AZRI), Bhakkar, Pakistan; 4 Department of Agronomy, Faculty of Agriculture and Environment, The Islamia University of Bahawalpur, Bahawalpur, Pakistan; 5 Department of Biology, Nakhchivan State University, Nakhchivan, Azerbaijan; 6 State Key Laboratory of Black Soils Conservation and Utilization, Northeast Institute of Geography and Agroecology, Chinese Academy of Sciences, Changchun, China; 7 Agricultural Biotechnology Department, College of Agriculture and Food Sciences, King Faisal University, Al-Ahsa, Saudi Arabia; 8 Department of Biology, College of Science, Princess Nourah Bint Abdulrahman University, Riyadh, Saudi Arabia; 9 Department of Agronomy, Kansas State University, Manhattan, KS, United States

**Keywords:** environmental sustainability, sowing method, soybean, weed competition, weed management

## Abstract

Soybean (*Glycine max* L.) yield is sensitive to early weed competition, yet optimal weed control timing and sowing methods remain unclear. Although early-season weed interference is widely recognized as detrimental, critical knowledge gaps persist regarding the precise weed-free period required and how sowing configuration affects weed dynamics and crop performance. Hence, this study aimed to clarify the effects of different weed-free durations and sowing methods (flat *vs*. bed) on soybean growth, yield, and seed quality. Over two years, a split-plot randomized block design with three replications was used to examine 16 treatments, including two sowing methods (bed sowing and flat sowing) with weed competition and weed-free periods of up to 25, 40, and 55 days after sowing (DAS), along with full-season competition and a weed-free period. Data were recorded to evaluate weed density, growth, yield, and quality parameters, including protein and oil content. Results showed that maintaining a weed-free window between 25–40 DAS was critical to minimizing yield losses. Weed competition throughout the season caused 57–62% yield loss in flat sowing and 58–60% in bed sowing, while weed-free management during 25–40 DAS maximized grain yield and improved seed protein and oil content. Flat sowing consistently outperformed bed sowing due to better canopy closure, reduced weed pressure, and improved resource capture during the critical weed-free period. In conclusion, maintaining a weed-free environment during the 25–40 DAS window, particularly under flat sowing, optimizes soybean growth, yield, and quality under non-GMO conditions. Future research should prioritize integrated, site-specific weed management strategies and assess their long-term impacts on economic returns, soil health, and environmental sustainability.

## 1 Introduction

Soybeans (*Glycine max* L.) are a crop of critical global importance due to their high nutritional value, which provides 18–22% oil and 40–42% protein, along with key vitamins and carbohydrates (Anwar et al., 2016). In countries like Pakistan, the increasing demand from the food, poultry, livestock, and marine industries has significantly escalated reliance on imported soybeans, oilseeds, and edible oils (Ren et al., 2021; Raza et al., 2021). This growing demand underscores the urgent need to expand domestic soybean production. However, soybean expansion and productivity in Pakistan and similar South Asian regions are constrained by several factors; however, weed infestation causes substantial yield losses in soybeans (Zhao et al., 2021a). Despite considerable progress in soybean breeding over the last two decades, inadequate weed control remains a primary constraint on yield improvement. Weed interference reduced soybean grain yield from ≈4.0 Mg ha^-1^ under season-long weeding to ≈1.3 Mg ha^-1^ under no-weeding (Caldas et al., 2023), while other field studies reported reductions from ~2.1–2.4 Mg ha^-1^ (weed-free) to 0.9–1.1 Mg ha^-1^ (no control) depending on environment and weed community (Daramola et al., 2019). Weeds not only outcompete soybeans for essential resources but also serve as alternate hosts for pests, insects, nematodes, and pathogens, compounding production costs and crop vulnerability (Imran et al., 2017; Arshad et al., 2025a, Arshad et al., 2026). The impact of weeds on yield depends on the weed species, density, emergence timing, and type (Zandoná et al., 2018; Bajwa et al., 2019; Saudy et al., 2020; Rizk et al., 2023). Soybean genotypes with longer growth durations and greater height have been linked to improved competitiveness, resulting in decreased weed seed production and a reduced size of weed species in the field (Saudy et al., 2020; El-Metwally and Saudy, 2021a). Notably, yield losses intensify with greater similarity between crop and weed traits, as competition for the same ecological niche reaches its maximum (El-Metwally et al., 2022; Saudy et al., 2022).

Weed pressure can arise at any stage of crop development (Mukherjee et al., 2012; Ishfaq et al., 2025), but the first six weeks after sowing represent the most critical period for yield loss, necessitating precise management during this phase. Many problematic weeds germinate before or synchronize their emergence with the crop, outpacing soybean growth, surpassing it in height, and intercepting photosynthetically active radiation through dense canopies and allelopathic effects (El-Metwally et al., 2021b; Keteku et al., 2020). Such competition can result in yield losses ranging from 39% to 87% (Mishra and Singh, 2009). Weeds accelerate stem elongation and decrease stem thickness in soybean, leading to lodging and reduced yield potential. When left uncontrolled, weeds are most aggressive early in the season, as soybean seedlings are weak competitors. As a result, weed pressure intensifies rapidly, and by four weeks after sowing, light competition becomes acute as weeds overshadow the crop (Mishra and Singh, 2009; El-Metwally and Saudy, 2021c; Saudy et al., 2022). Weed infestation during the early growth stages of soybean can significantly reduce seed yield and quality, particularly protein and oil content (Gawęda et al., 2024; Petcu et al., 2023; OReilly et al., 2025). Studies consistently show that heavy weed pressure leads to lower protein and fat content in seeds, while effective weed control (herbicides, hand weeding, or integrated methods) improves these parameters. The critical period for weed competition is typically the first 30 days after sowing (Morsy and Tantawy, 2018).

The impact of weeds on soybean follows consistent physiological principles across environments, as soybean growth stages and competitive responses are largely uniform globally. Studies from diverse regions report similar patterns: weeds interfere most severely during the early vegetative phase through early reproductive development, commonly referred to as the critical weed-free period (CWFP) (Trisnaningsih et al., 2024; Rizk et al., 2023). During this period, uncontrolled weeds reduce light interception, nutrient uptake, and canopy development, ultimately resulting in substantial yield losses. Timely weed management at these stages is associated with reductions in weed density and improved yield (Rüdell et al., 2021; da Silva et al., 2021). Although individual studies report different numerical ranges for the CWFP, these differences mainly reflect local weed flora, management practices, and environmental conditions rather than fundamental differences in soybean physiology. Overall, the literature consistently identifies early-season weed control as essential for minimizing yield loss and maintaining crop productivity (Mubeen et al., 2021; Absy and Yacoub, 2020).

Sowing method influences both weed infestation and seed quality. Bed sowing and narrow row spacing generally reduce weed density and biomass, leading to higher yields and improved seed quality (Gawęda and Kopcewicz, 2023; Arshad et al., 2026). Broad bed furrow sowing, has been shown to minimize weed density and maximize yield and economic returns 27. However, some studies report minimal or inconsistent effects of sowing method on seed protein and oil content, suggesting that environmental factors and cultivar choice also play significant roles (Singh et al., 2023; Štefanić et al., 2022);. Land management and sowing configurations strongly influence soybean performance. Recently adopted methods, such as ridge, flat, and particularly wide-bed and furrow sowing, have demonstrated benefits for soil aeration, moisture conservation, and temperature regulation (Keteku et al., 2020). Bed-furrow systems have been shown to enhance growth and productivity by reducing seed rates and improving moisture management, particularly under rainfed conditions (Keteku et al., 2020; Sharma et al., 2020). In contrast, flat-bed sowing can increase the risk of waterlogging, soil loss, and reduced moisture retention during dry spells, as observed in Vertisols (Sharma et al., 2020). Broad-bed and furrow systems are particularly recommended for watershed development and mitigating the effects of excess rainfall (Keteku et al., 2020). Globally, uncontrolled weed competition can reduce soybean yields by as much as 80% (Daugovish et al., 2003). While herbicide-resistant cultivars and widespread herbicide use underpin weed management in major soybean-producing countries, regulatory constraints in Pakistan prohibit the use of genetically modified (GMO) herbicide-resistant soybeans. As such, the reliance on non-GMO varieties necessitates alternative approaches to weed control, further amplifying the urgency to develop sustainable, localized weed management strategies that can minimize yield losses and control costs (Trisnaningsih et al., 2024; Arshad et al., 2025b). Meta−analytical evidence also supports the role of agronomic practices in weed suppression: a recent meta-analysis (Singh et al., 2023) found that narrow row spacing (< 76 cm) in soybean significantly reduced weed density (by ~34%) and improved weed control by ~32%, ultimately contributing to yield stability under competitive conditions. Integrated approaches—combining optimal sowing methods with effective weed control—yield the best results for seed quality and yield (OReilly et al., 2025). Pre-emergence herbicide application in bed or narrow-row sowing systems can significantly enhance protein content and yield (Gawęda et al., 2024; Petcu et al., 2023). Conversely, poor weed control or suboptimal sowing methods can negate potential benefits (Haliniarz et al., 2017; Štefanić et al., 2022).

An improved understanding of weed emergence dynamics is crucial, as the timing and pattern of weed emergence critically affect crop–weed competition and associated yield loss (Dorado et al., 2009). Despite decades of research on weed modelling and prediction, knowledge gaps remain regarding optimal strategies for integrating weed control with land preparation and sowing methodology under site-specific conditions. The central challenge for sustainable soybean cultivation in Pakistan lies in determining how weed management efficacy depends on sowing method (flat versus bed) and the timing/duration of both weed infestation and weed removal (Safdar et al., 2020). Despite an extensive global literature on weed-crop competition and the critical periods for weed control in soybean, most studies have focused on herbicide-resistant cultivars or have been conducted in environments with different soil types, climates, and management practices than those prevalent in Pakistan (Daugovish et al., 2003; Dorado et al., 2009; Arshad et al., 2025b). In Pakistan, the exclusive cultivation of non-GMO soybean varieties and limited access to herbicide-based weed control highlight the need for site-specific, integrated management strategies. Furthermore, while the negative impact of early-season weed interference and the potential benefits of various sowing configurations are recognized, there is limited knowledge on how the interaction between sowing methods (flat *vs*. bed) and the timing and duration of weed control can be optimized to improve yield and resource-use efficiency under local, non-GMO production systems.

To address this research gap, the present study aimed to evaluate the effects of different weed-free durations and sowing methods on soybean growth, yield, and seed quality. Specifically, this work: (1) assessed how different sowing methods (flat *vs*. bed sowing) influence weed emergence and crop performance, (2) determined the most critical periods when weed control is necessary to prevent significant yield loss, and (3) sought to identify practical, resource-efficient weed management strategies for sustainable soybean production in non-GMO systems. It was hypothesized that the efficacy of weed management in soybeans was significantly shaped by both the sowing method and the precise duration of weed interference and control. Integrating optimal sowing techniques with the identification of critical weed-free periods would, therefore, significantly enhance soybean yield and quality by minimizing weed-related losses and better aligning management interventions with local production realities.

## 2 Materials and methods

### 2.1 Study area description

**Figure 1:**
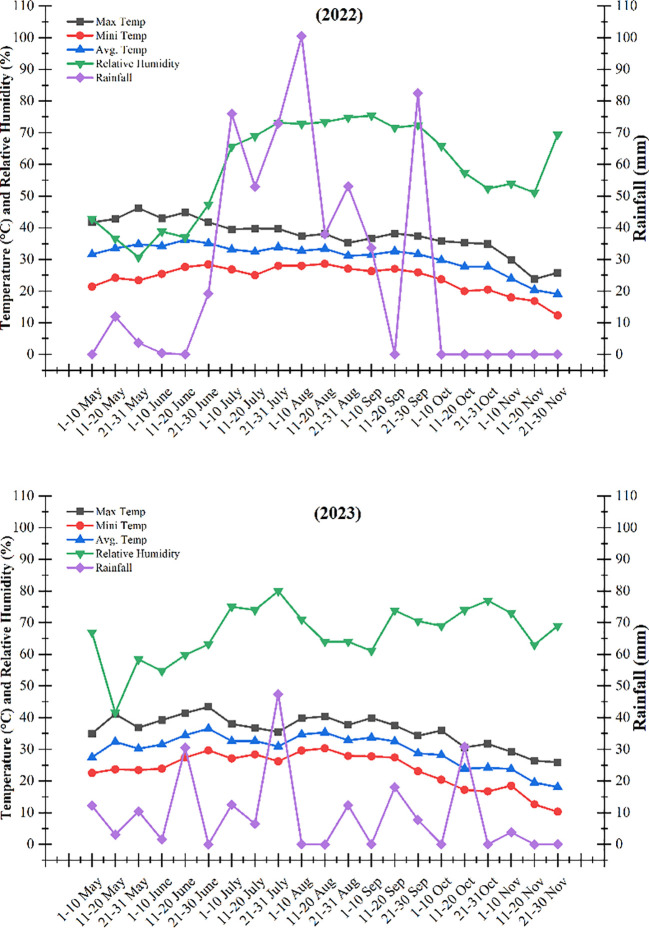
The maximum, minimum, and average temperature (°C), relative humidity (%), and rainfall (mm) during the soybean growing seasons.

The field trials were carried out during the 2022 and 2023 growing seasons at the Agronomic Research Farm, Department of Agronomy, University of Agriculture, Faisalabad. The site is located at 31.4504° N latitude, 73.1350° E longitude, and an elevation of 186.4 m above sea level. The soil at the site was classified as sandy clay loam in texture, with a pH of 7.7 in 2022 and 7.5 in 2023. Soil chemical properties, including total nitrogen (0.13% and 0.16%) and organic matter content (0.89% and 0.95%), were determined from composite soil samples collected from the 0–30 cm soil layer for the two respective years. Weather conditions varied noticeably between the two growing seasons at the Agronomic Research Farm, University of Agriculture, Faisalabad, Pakistan (**Figure 1**). The 2022 season received comparatively higher and more evenly distributed rainfall than 2023, whereas rainfall in 2023 was lower and more erratic, particularly during the early crop establishment period. Mean air temperatures and relative humidity followed a broadly similar seasonal pattern in both years, although short-term fluctuations were observed. These interannual differences in rainfall and temperature likely influenced weed emergence dynamics, herbicide efficacy, and overall soybean performance.

### 2.2 Experimental design and treatments

The field experiment was conducted in a split-plot arrangement under a randomized complete block design (RCBD) with three replications. A total of 16 treatments were employed and arranged in a randomized complete block design with a split-plot structure, each treatment replicated three times to ensure robust statistical analysis. The treatments consisted of two sowing methods (Flat sowing and bed sowing) with four weed-competition periods (25, 40, 55 DAS, & whole season) and four weed-free periods (25, 40, 55 DAS, & whole season). Each treatment was replicated three times, resulting in 48 experimental plots. Each experimental plot measured 6 m × 2.1 m (12.6 m²), and the total gross area of the field experiment was 604.8 m². The net harvested area for yield determination was 5 m × 1.5 m (7.5 m²), obtained by excluding the outer border rows and 0.5 m from each end of the plot to minimize border effects. The defined weed-competition and weed-free durations were based on soybean phenological development according to the Fehr and Caviness soybean growth stage scale. Specifically, 25 DAS corresponded to the early vegetative stages V2–V3 (second to third trifoliate leaf fully expanded), 40 DAS to vegetative branching stages V4–V5 (fourth to fifth trifoliate leaf), and 55 DAS coincided with early reproductive stages R1–R2 (beginning bloom to full bloom) in soybean (Fehr and Caviness, 1977). Weed control during the designated periods was carefully maintained through weekly hand-weeding whenever necessary.

### 2.3 Sowing methods

Soybean variety AARI (seed source: Ayub Agricultural Research Institute, Faisalabad) was used in this study. This cultivar is a locally released material adapted to the agro-climatic conditions of central Punjab and is commonly used in research and farmers’ fields around Faisalabad. It is characterized as a medium-maturity type under local conditions (commonly classified in the region as approximately 110–120 days to physiological maturity), with a determinate-to-semi-determinate growth habit and moderate lodging resistance. Previous varietal evaluations of AARI-released and locally adapted soybean cultivars in Pakistan report mean seed protein contents ranging from approximately 38–42% and oil (fat) contents ranging from 18–21%, indicating suitability for both food and feed purposes (Asad et al., 2020). Reported 100-seed weight for locally adapted soybean varieties and lines in the region typically ranges between ~11 and 16 g; the cultivar AARI has been reported among the better-adapted, moderate-seed-weight materials in previous local evaluations. In flat sowing, soybean was sown using a manual hand drill in rows spaced 30 cm apart with plant-to-plant spacing of 7 cm with a hand drill, using a seeding rate of 100 kg ha^-1^. This drilling method ensures uniform seed placement, optimal plant population, and better weed management through mechanical inter-row cultivation. In bed sowing, soybean seeds were placed manually using a hand-operated single-row seed drill adapted for raised beds, following the ridge-and-furrow system with beds 60 cm apart (furrow to furrow). Two rows of soybeans were planted on each bed, 30 cm apart, and 7 cm between plants, maintaining the same seeding rate of 100 kg ha^-1^. This method improves water-use efficiency, reduces weed infestation in furrows, and enhances soil aeration and root growth, thus contributing to sustainable cultivation under varying agro-climatic conditions. These methods were selected based on regional agronomic guidelines and to assess their potential in enhancing resource-use efficiency and suppressing weed growth under different spatial geometries, thereby promoting sustainable soybean production.

### 2.4 Data collection

Data were recorded for total dry matter and leaf area index to evaluate soybean growth. Additionally, weed density, weed dry weight, and soybean yield were measured. Seed oil and protein content were also analyzed as indicators of quality. Each plot was split into two subplots: one for growth analysis and the other for final yield. Growth measurements were taken destructively from a 1 ft^2^ sampling area, later converted to a per-square-meter basis. Sampling was conducted at 30, 45, 60, 75, and 90 days after sowing (DAS). To calculate LAI, leaves were detached and weighed fresh. From each sample, a 10 g fresh leaf subsample was taken to determine leaf area using a LI-3100C leaf area meter. The specific leaf area (SLA, cm^2^ g^-1^) was calculated as the leaf area per unit dry weight, and total plot-level leaf area was then estimated by multiplying the SLA by total leaf biomass. This approach conforms to standard protocols outlined by Hunt (1990). All plant material was first sun-dried to reduce moisture, followed by oven drying at 65 ± 2 °C until a constant weight was achieved for dry matter determination.

### 2.5 Growth parameter measurement

#### 2.5.1 Crop vigor score

Crop vigor was visually assessed at 105 DAS using a 0–10 scale, where 0 denoted completely dead or very weak plants and 10 represented the most vigorous stands, following the method described by Nikoa et al. (2015). The crop growth rate (CGR) was calculated using the standard formula that measures the increase in dry matter over a given time period:


$$CGR=\frac{W_{2\;}-\;W_{1}}{t2-t1}$$

In this formula, W1 and W2 denote the dry weights recorded at two different intervals, T1 (initial measurement at 30 DAS) and T2 (subsequent measurement taken after 15 days). The leaf area index (LAI), a key indicator of growth, was determined using the following equation.


$$\text{LAI }=\;\frac{leaf\;\;area}{land\;\;area}$$

Leaf area duration (LAD) was computed using the leaf area indices recorded at two stages, where LAI1 and LAI2 corresponded to measurements taken at times t1 and t2, respectively.


$$LAD=\frac{\left(LAI1+LAI2\right)\left(t2-t1\right)}{2}$$

Leaf area per plant was estimated using ten randomly tagged plants from each treatment. For each plant, the length (L) and width (W) of the terminal leaflet were measured, and their product was multiplied by a correction factor to account for leaf shape. From these measurements, the leaf area index (LAI) was derived, providing an important measure of canopy development and soybean growth. Net assimilation rate (NAR), which reflects the gain in dry matter per unit leaf area after accounting for respiratory losses, was then calculated.


$$NAR=\frac{TDM}{LAD}$$

### 2.6 Weed assessment

The weed cover score is a visual observation conducted before weed removal and evaluated on a scale of 1 to 10, where 1 represents a completely weed-free situation and 10 represents complete weed coverage, following the scale described by Adigun et al. (2017). Weed density was recorded at 25, 40, and 55 DAS and at harvest by counting the number of weed species within a 1 m^-2^ quadrat placed randomly at three locations within each plot at 25, 40, 55 DAS, and at harvest. Weeds were harvested by cutting them at ground level, oven-dried at 70 °C for 72 hours, and their dry weight was recorded in grams per square meter (g m^-2^). Weed indices were also recorded, including weed control efficiency and other standard procedures as follows: The weed control efficiency (WCE) is calculated by assessing the decrease in dry weight of weeds in treated plots in comparison to the dry weight of weeds in untreated or control plots (Rana and Kumar, 2014; Rana and Rana, 2016; Rana, 2016). It’s also expressed as a percentage. where: WC = Weed dry weight in the control (unweeded) plot. WT = Weed dry weight in treated plot.


$$\text{WCE}=\frac{{\text{W}}_{C}-{\text{W}}_{\text{T}}}{\;\;\;{\text{W}}_{\text{C}}}\times \;100$$

To calculate the weed index (WI), we use two values: the yield from a plot without weeds (YWF) and the yield from the treated plot (YT) (Kropff and Spitters, 1991), We use a simple formula to find the WI:


$$\text{WI}=\frac{{\text{Y}}_{\text{WF}}-{\text{Y}}_{\text{T}}}{\;\;\;{\text{Y}}_{\text{WF}}}\;\times 100$$

To calculate the weed persistence index (WPI), we use four values: the weed dry weight in the control (unweeded) plot (WC), the weed dry weight in the treated plot (WT), the weed population in the control (unweeded) plot (WPC), and the weed population in the treated plot (WPT) (Rana and Rana, 2016; Rana, 2016). The formula is as follows:


$$\text{WPI}=\frac{{\text{W}}_{\text{T}}}{{\text{W}}_{\text{C}}}\times \;\frac{{\text{W}}_{\text{PC}}}{{\text{W}}_{\text{pT}}}$$

### 2.7 Statistical analysis

All recorded data were organized and analyzed using Statistix 8.1. Prior to analysis, the assumptions of ANOVA were verified. Normality of the data distribution was assessed using the Shapiro–Wilk test and by visual inspection of residual normal probability plots, while homogeneity of variances was tested using Levene’s test. Treatment means were compared after performing Fisher’s Analysis of Variance (ANOVA), and significant differences were identified with the Least Significant Difference (LSD) test at the 5% probability level (P ≤ 0.05). Relationships between sowing methods and periods of weed competition, as well as their impact on growth and yield, were also examined. Proper randomization and replication in the experimental design ensured the reliability and validity of the statistical outcomes. Graphical illustrations were prepared using Origin 2024b. Linear regression was performed in R-Statistical package and visualize in ggplot2.

## 3 Results

### 3.1 Dominant weed flora and infestation trends

The study revealed that *Trianthema portulacastrum* L. and *Convolvulus arvensis* L. (both broadleaf weeds, at 60–90% infestation levels) and *Cynodon dactylon* L. (grass, at 60–90% infestation levels) consistently showed high infestation levels in both 2022 and 2023. *Tribulus terrestris* L. (broadleaf weed) infestation dropped from high (60–90%) to moderate (30–59%) over the two years. Additionally, *Euphorbia granulate* L. and *Parthenium hysterophorus* L. (both broadleaf weeds) saw decreases in infestation levels, with *Cyperus rotundus* L. (sedge) remaining highly infested (60–90%) (**Supplementary Table S1**).

### 3.2 Weed cover score, density, and dry weight

Weed cover score, weed density, and weed dry weight were markedly affected by the combination of sowing method and weed management regime (**Table 1**). The highest weed cover scores and densities were observed in plots under bed sowing with a full-season weed competition period, reaching 8.9 and 90.3 in 2022, and 9.0 and 83.0 in 2023, respectively. In contrast, the lowest values for these parameters were recorded in flat sowing with a full-season weed-free regime (2.1 and 16.7 in 2022; 1.5 and 10.7 in 2023). Intermediate weed densities were observed in both sowing methods when weed-free periods extended only up to 25 days after sowing (DAS), indicating that early weed removal is effective but less so than maintaining weed-free conditions throughout the season. Notably, the higher weed densities and cover scores observed in 2022 compared with 2023 may be attributed to relatively higher rainfall during the early crop growth stages in 2022, which likely promoted weed emergence, whereas the slightly warmer and drier conditions in 2023 may have limited weed establishment. Patterns in weed dry weight (kg ha^-^¹) mirrored these observations. Bed sowing with full-season competition yielded the greatest weed biomass (4346.2 kg ha^-1^ in 2022 and 4186.6 kg ha^-1^ in 2023), whereas the lowest dry weights occurred under flat sowing with a full-season weed-free period (1174.8 kg ha^-1^ in 2022 and 982.1 kg ha^-1^ in 2023). Transitioning from continuous competition to season-long weed-free management resulted in dramatic reductions in weed pressure. Under bed sowing, weed cover scores decreased by approximately 76% (from 8.9 to 2.1 in 2022, and from 9.0 to 1.5 in 2023); weed density declined by 81% (from 90.3 to 16.7 in 2022 and from 83.0 to 10.7 in 2023); and weed dry weight was reduced by 73% (from 4346.2 to 1174.8 kg ha^-1^ in 2022) and 77% (from 4186.6 to 982.1 kg ha^-1^ in 2023). These findings underscore the substantial benefit of maintaining a weed-free environment through the entire growing season. Collectively, the results demonstrate that flat sowing combined with season-long or at least early weed-free management substantially reduces weed infestation, as evidenced by lower weed cover, density, and dry biomass, thereby improving the potential for enhanced soybean productivity.

**Table 1:** Weed cover score (0–10 scale), weed density (plants m^-^²), and dry weight (kg ha^-^¹) as influenced by sowing method and period of weed interference in soybean in 2022 and 2023.

Treatments		Weed cover score (0–10 scale)		Weed Density (plants m^-2^)		Weed Dry weight (kg ha^-^¹)	
		2022	2023	2022	2023	2022	2023
Sowing method	Competition period						
Flat Sowing	Competition upto 25 DAS	3.8 ± 0.13^h^	2.5 ± 0.09^k^	45.67 ± 0.33^i^	39.33 ± 0.88^h^	2824.36 ± 5.82^h^	2543.97 ± 18.22^h^
	Competition upto 40 DAS	5.2 ± 0.07^f^	3.6 ± 0.06^j^	66.0 ± 0.58^d^	54.67 ± 0.88^e^	3782.61 ± 8.49^f^	3328.63 ± 9.79^e^
	Competition upto 55 DAS	6.1 ± 0.09^d^	6.6 ± 0.07^e^	67.0 ± 0.58^d^	65.00 ± 0.58^d^	3853.32 ± 12.99^e^	3545.73 ± 11.27^d^
	Full season competition period	8.7 ± 0.06^a^	8.1 ± 0.07^c^	78.7 ± 0.33^b^	76.67 ± 0.88^b^	4131.70 ± 11.30^b^	4054.41 ± 14.17^b^
	Weed free upto 25 DAS	6.6 ± 0.09^c^	7.3 ± 0.11^d^	41.0 ± 0.58^k^	28.67 ± 0.88^k^	2726.24 ± 9.95^i^	2328.98 ± 10.80^i^
	Weed free upto 40 DAS	5.5 ± 0.08^e^	6.4 ± 0.09^f^	43.7 ± 0.33^j^	35.67 ± 0.33^i^	2631.94 ± 11.82^j^	1864.62 ± 6.73^l^
	Weed free upto 55 DAS	4.1 ± 0.09^g^	5.1 ± 0.08^h^	40.0 ± 0.58^k^	33.33 ± 0.67^j^	2054.74 ± 16.57^m^	1725.59 ± 7.27^m^
	Full season weed free period	2.1 ± 0.03^j^	1.5 ± 0.08^m^	16.7 ± 0.33^m^	10.67 ± 0.88^m^	1174.76 ± 11.91°	982.13 ± 10.22°
Bed Sowing	Competition upto 25 DAS	4.2 ± 0.10^g^	3.7 ± 0.08^j^	55.0 ± 0.58^e^	50.00 ± 0.58^f^	3048.58 ± 8.20^g^	2974.67 ± 6.49^f^
	Competition upto 40 DAS	6.3 ± 0.08^d^	4.6 ± 0.09^i^	75.0 ± 0.58^c^	66.00 ± 0.58^d^	3963.85 ± 12.20^d^	3528.15 ± 7.95^d^
	Competition upto 55 DAS	7.6 ± 0.09^b^	8.6 ± 0.08^b^	77.0 ± 0.58^b^	72.00 ± 0.58^c^	4064.63 ± 12.14^c^	3781.49 ± 6.23^c^
	Full season competition period	8.9 ± 0.06^a^	9.0 ± 0.04^a^	90.3 ± 0.33^a^	83.00 ± 0.58^a^	4346.17 ± 20.25^a^	4186.57 ± 8.17^a^
	Weed free upto 25 DAS	7.3 ± 0.09^b^	7.3 ± 0.10^d^	53.0 ± 0.58^f^	41.00 ± 0.58^h^	2745.61 ± 17.33^i^	2650.14 ± 7.30^g^
	Weed free upto 40 DAS	6.2 ± 0.16^d^	6.5 ± 0.10^ef^	51.0 ± 0.58^g^	44.00 ± 0.58^g^	2462.25 ± 11.33^k^	2172.57 ± 6.40^j^
	Weed free upto 55 DAS	5.4 ± 0.06^ef^	6.1 ± 0.06^g^	49.0 ± 0.58^h^	40.00 ± 0.58^h^	2254.22 ± 15.59^l^	2057.76 ± 6.66^k^
	Full season weed free period	2.4 ± 0.09^i^	2.1 ± 0.09^l^	25.0 ± 0.58^l^	17.33 ± 0.88^l^	1456.82 ± 19.89^n^	1250.63 ± 6.46^n^
	MS for sowing methods (S)	6.9769**	8.4588**	1102.1**	901.33**	253380**	930690**
	MS for competition period (CP)	24.3807**	33.5344**	16923.3**	2672.38**	6034583**	6127443**
	MS for S × CP	0.3015**	0.6098**	26.6**	8.76**	32867**	12495**
	LSD At 5%	0.2610	0.2218	1.8076	2.1764	54.005	27.940

Error bars represent the standard deviation of three biological replicates. Bars (or points) sharing different lowercase letters are significantly different according to *post hoc* tests (*P<* 0.05).

### 3.3 Weed indices analysis

**Figure 2:**
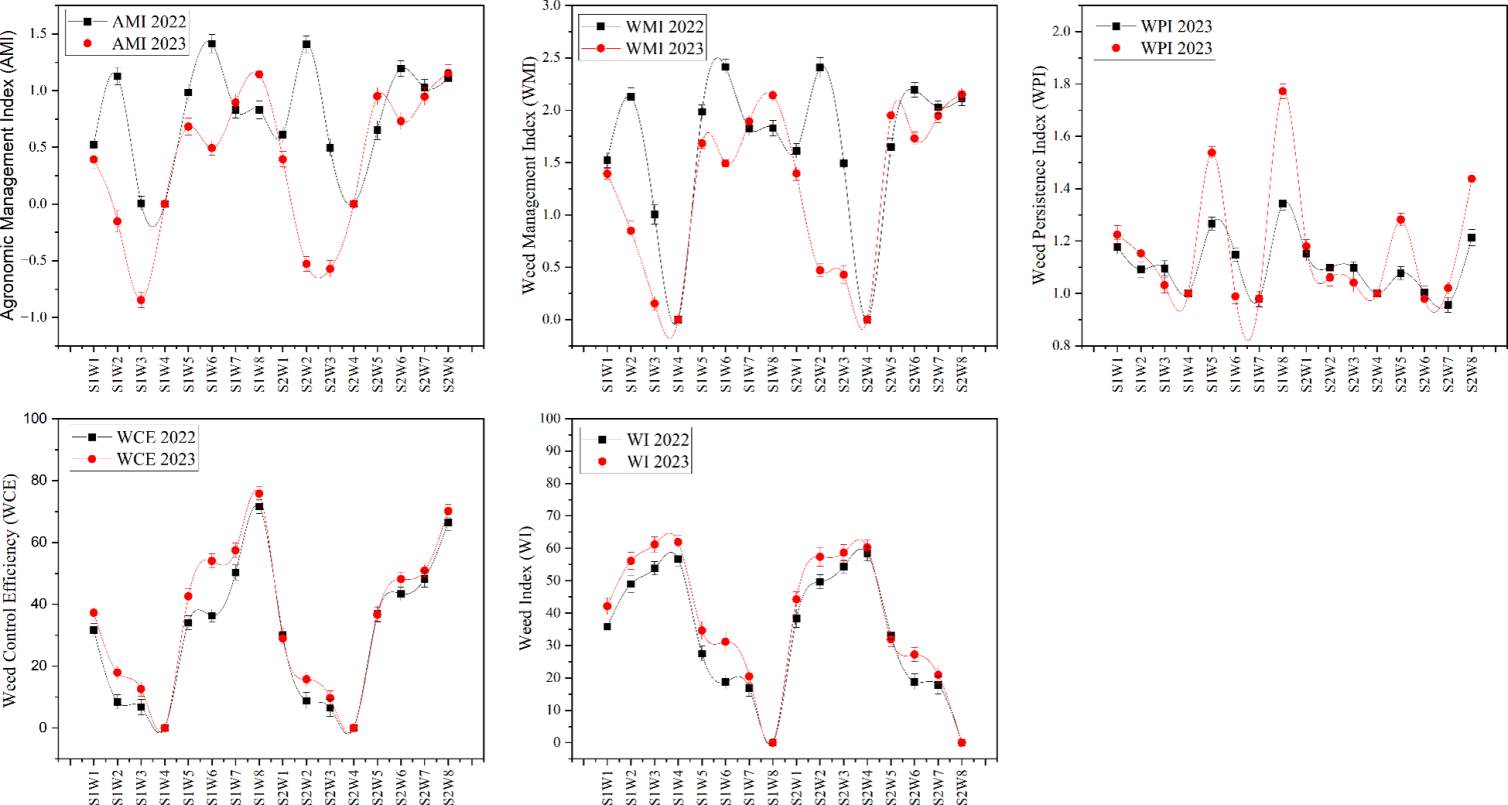
Effects of sowing method and weed competition period on weed indices in soybean over two seasons. Weed indices analyzed include the weed index (WI), weed control efficiency (WCE), weed persistence index (WPI), weed management index (WMI), and agronomic management index (AMI). S1: Flat sowing; S2: Bed sowing. Weed management treatments: W1, Competition up to 25 days after sowing (DAS); W2, Competition up to 40 DAS; W3, Competition up to 55 DAS; W4, Full-season competition; W5, Weed-free up to 25 DAS; W6, Weed-free up to 40 DAS; W7, Weed-free up to 55 DAS; W8, Full-season weed-free. Error bars represent the standard deviation of three biological replicates. Bars (or points) sharing different lowercase letters are significantly different according to *post hoc* tests (P< 0.05).

Assessment of multiple weed indices, including the weed index (WI), weed control efficiency (WCE), weed persistence index (WPI), weed management index (WMI), and agronomic management index (AMI), across two seasons revealed clear impacts of sowing method and weed management duration (**Table 2**; **Figure 2**). In both years, WI varied significantly in response to both sowing configuration and the length of weed competition. Full-season weed competition consistently produced the highest WI values, with losses reaching 56.7–61.9% in flat sowing and 58.4–60.3% in bed sowing, emphasizing the extent of yield reduction under unmanaged conditions. The lowest WI values were recorded when fields were kept weed-free up to 25, 40, or 55 DAS, with timely weed control leading to substantially improved crop performance. The data underscore that keeping fields weed-free between 25 and 55 DAS is particularly effective in lowering WI and associated yield penalties.

**Table 2:** Soybean growth response to sowing method and different period of weed interference in 2022 and 2023.

Treatments		Crop vigor score		Cumulative leaf area duration		Net assimilation rate	
		2022	2023	2022	2023	2022	2023
Sowing method	Competition period						
Flat Sowing	Competition upto 25 DAS	5.2 ± 0.07^e^	7.5 ± 0.03^i^	165.36 ± 0.10^e^	163.55 ± 0.18^g^	1.95 ± 0.00^g^	2.07 ± 0.01^h^
	Competition upto 40 DAS	5.0 ± 0.09^fg^	6.5 ± 0.04^k^	160.85 ± 0.06^f^	156.39 ± 0.30^h^	1.97 ± 0.00^g^	2.04 ± 0.01^i^
	Competition upto 55 DAS	5.2 ± 0.05^ef^	6.1 ± 0.05^m^	142.67 ± 0.20^h^	145.62 ± 0.81^j^	1.73 ± 0.01^h^	1.71 ± 0.01^l^
	Full season competition period	4.2 ± 0.04^h^	5.1 ± 0.04^f^	126.52 ± 0.72^j^	127.45 ± 0.45^l^	1.46 ± 0.01^j^	1.36 ± 0.01^m^
	Weed free upto 25 DAS	5.2 ± 0.03^cd^	7.4 ± 0.04^h^	179.38 ± 0.47^d^	182.66 ± 0.78^d^	2.51 ± 0.00^bcd^	2.33 ± 0.01^f^
	Weed free upto 40 DAS	5.6 ± 0.04^bcd^	7.5 ± 0.01^e^	189.70 ± 0.71^c^	195.63 ± 0.37^c^	2.51 ± 0.01^bcd^	2.47 ± 0.01^d^
	Weed free upto 55 DAS	6.1 ± 0.06^bc^	7.7 ± 0.03^c^	210.37 ± 0.48^b^	211.67 ± 0.59^b^	2.53 ± 0.01^bc^	2.41 ± 0.01^e^
	Full season weed free period	6.7 ± 0.05^a^	7.8 ± 0.02^a^	224.50 ± 0.63^a^	227.36 ± 0.75^a^	2.66 ± 0.01^a^	2.60 ± 0.01^a^
Bed Sowing	Competition upto 25 DAS	5.2 ± 0.05^ef^	7.1 ± 0.06^j^	149.90 ± 1.19^g^	149.45 ± 0.34^i^	2.20 ± 0.01^e^	2.08 ± 0.00^h^
	Competition upto 40 DAS	5.2 ± 0.03^g^	6.5 ± 0.02^l^	139.51 ± 0.15^i^	141.28 ± 0.48^k^	2.08 ± 0.01^f^	2.15 ± 0.00^g^
	Competition upto 55 DAS	5.1 ± 0.06^h^	6.5 ± 0.02^n^	125.30 ± 1.12^j^	128.00 ± 0.51^l^	1.67 ± 0.01^i^	1.87 ± 0.01^j^
	Full season competition period	4.1 ± 0.07^h^	6.0 ± 0.00°	105.06 ± 0.29^k^	106.74 ± 0.50^m^	1.70 ± 0.01^hi^	1.76 ± 0.00^k^
	Weed free upto 25 DAS	5.4 ± 0.03^d^	7.4 ± 0.02^g^	163.75 ± 0.31^ef^	165.82 ± 0.67^f^	2.51 ± 0.00^cd^	2.50 ± 0.01^c^
	Weed free upto 40 DAS	6.1 ± 0.07^cd^	7.5 ± 0.04^g^	178.57 ± 0.16^d^	179.42 ± 0.45^e^	2.64 ± 0.00^a^	2.57 ± 0.00^b^
	Weed free upto 55 DAS	6.3 ± 0.03^bc^	7.5 ± 0.04^d^	192.56 ± 2.06^c^	194.18 ± 0.22^c^	2.54 ± 0.03^b^	2.60 ± 0.00^a^
	Full season weed free period	6.4 ± 0.03^b^	7.7 ± 0.00^a^	210.20 ± 0.16^b^	210.93 ± 0.31^b^	2.48 ± 0.00^d^	2.58 ± 0.01^b^
	MS for sowing methods (S)	0.07521^ns^	0.12000**	3391.42**	3392.00**	0.04642*	0.23115**
	MS for competition period (CP)	3.47116**	3.79857**	6959.04**	7130.59**	0.99851**	0.85658**
	MS for S × CP	0.10807**	0.30143**	18.28**	5.80**	0.03429**	0.02426**
	LSD At 5%	0.3704	0.0280	3.0593	1.8564	0.0445	0.0224

Error bars represent the standard deviation of three biological replicates. Bars (or points) sharing different lowercase letters are significantly different according to *post hoc* tests (*P<* 0.05).

The evaluation of WCE further illustrated the influence of both sowing methods and weed-free intervals. Flat sowing paired with weed-free periods of 40–55 DAS produced the greatest WCE (40–60%), while bed sowing under similar competition durations recorded the lowest (10–15%). This trend demonstrates that not only the timing but also the method of sowing strongly affects weed control efficacy. Similar trends were observed in WPI: for flat sowing, WPI declined from 1.18 at 25 DAS to 1.10 at 55 DAS, while bed sowing followed a comparable pattern, with a modest rise in 2023 but overall temporal consistency.

### 3.4 Crop growth parameters

**Figure 3:**
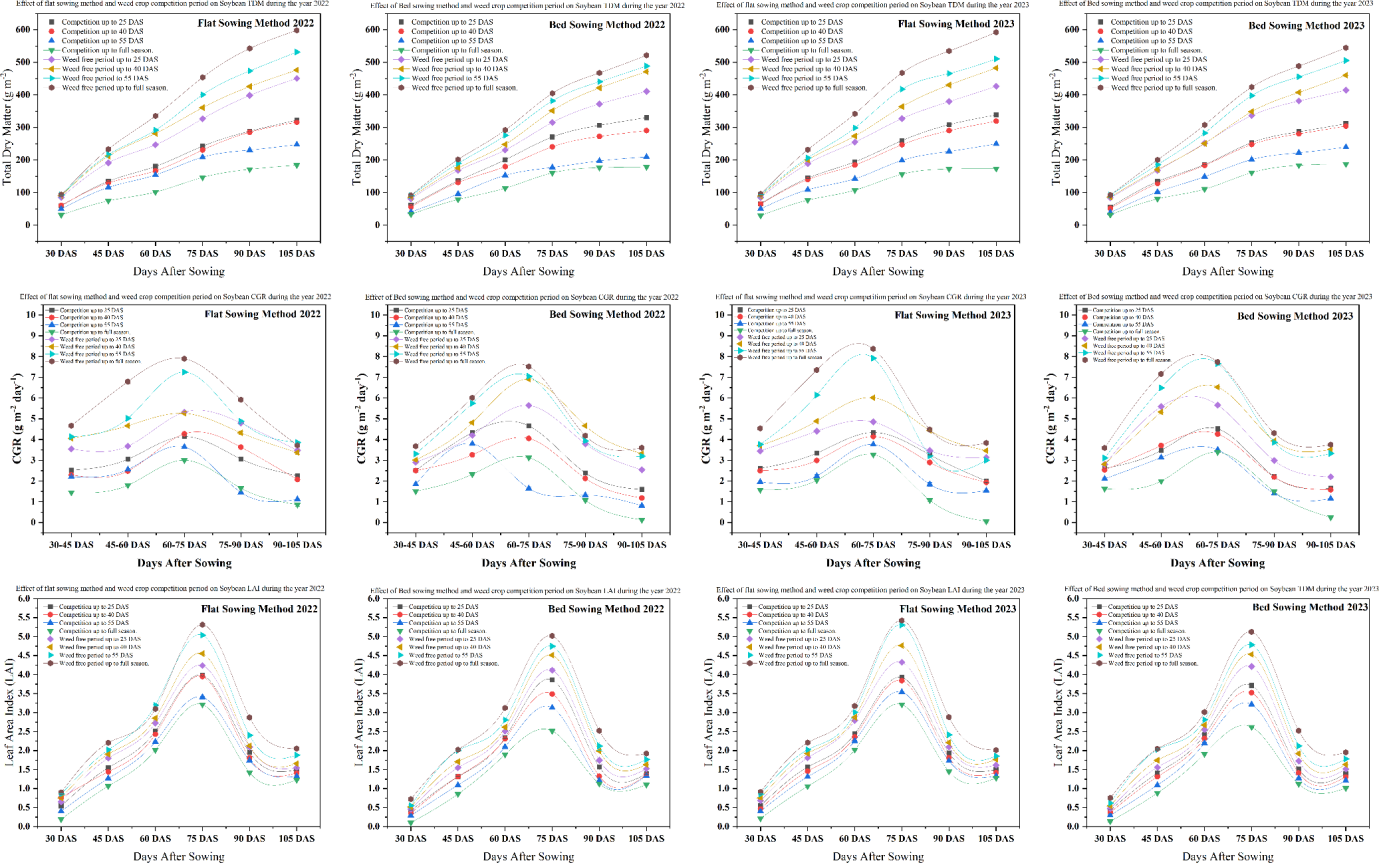
Effect of sowing methods and weed crop competition period on soybean total dry matter, crop growth rate, leaf area index during the year 2022 and 2023. Error bars represent the standard deviation of three biological replicates. Bars (or points) sharing different lowercase letters are significantly different according to *post hoc* tests (*P<* 0.05).

Across both 2022 and 2023, total dry matter (TDM) production showed a strong dependence on sowing method and the duration of weed interference (**Figure 3**). Under flat sowing in 2022, maximum TDM was recorded when plots were maintained weed-free up to 40 DAS (521.3 g m^-^²), whereas full-season weed competition drastically reduced TDM to only 101.5 g m^-^². A similar pattern was observed in 2023, highlighting the consistent advantage of early and sustained weed control. In bed sowing, TDM was likewise higher under weed-free conditions, peaking at 491.1 g m^-^² in 2022 and 490.5 g m^-^² in 2023 under weed-free up to 40 DAS, while full-season competition reduced TDM to 130.9–184.3 g m^-^². These results confirm that early and effective weed management is critical for maximizing soybean biomass accumulation, irrespective of the sowing method employed.

Leaf area index (LAI) followed trends similar to TDM. Under flat sowing, LAI values at 30 DAS were comparable for treatments with weed-free or competition periods up to 25 DAS (0.54 and 0.65 in 2022), but diverged substantially thereafter. The highest LAI was observed under weed-free conditions up to 55 DAS (5.04 in 2022 and 5.31 in 2023), whereas full-season weed competition restricted LAI to 1.23–1.88 in 2022 and 1.28 in 2023. In bed sowing, peak LAI was also recorded under weed-free up to 55 DAS (3.10 in 2022; 4.78 in 2023), while the lowest values were associated with full-season competition (1.10 in 2022; 1.01 in 2023). Overall, LAI values were higher in 2023, reflecting more favorable growing conditions and reinforcing the role of weed suppression in promoting canopy development.

Crop growth rate (CGR) mirrored the responses of both TDM and LAI. In flat sowing, CGR peaked at 7.25 g m^-^² d^-^¹ in 2022 and 8.37 g m^-^² d^-^¹ in 2023 under weed-free conditions during the 60–75 DAS interval, compared with 4.27 and 4.34 g m^-^² d^-^¹, respectively, under weed competition. Although bed sowing generally exhibited lower CGR, the same pattern was maintained, with maximum rates always associated with weed-free treatments. These observations highlight the strong suppressive effect of prolonged weed interference on crop growth dynamics.

Crop vigor further reflected the superiority of weed-free conditions across both sowing methods (**Table 2**). In 2022, the highest vigor scores were recorded under full-season weed-free conditions (6.7 in flat sowing and 6.4 in bed sowing), whereas full-season weed competition reduced these values to 4.2 and 4.1, respectively. A similar trend was evident in 2023. Cumulative leaf area duration (LAD) also increased markedly under weed-free conditions. In 2022, flat sowing maintained weed-free throughout the season recorded the highest LAD (224.5) compared to only 126.5 under full-season competition, with extended weed-free periods up to 55 DAS consistently outperforming competitive treatments. These patterns were consistent in 2023, confirming the stability of this response across seasons.

Net assimilation rate (NAR) responded positively to prolonged weed-free conditions. In 2022, the highest NAR values (2.53–2.66) were observed in flat sowing maintained weed-free up to 55 DAS and throughout the season, whereas lower values (1.67–1.70) were recorded under full-season competition, particularly in bed sowing. Similar rankings were observed in 2023. Collectively, these findings demonstrate that crop vigor and key physiological attributes, including LAI, LAD, CGR and NAR, are maximized when soybean is kept weed-free during the critical early to mid-growth stages (40–55 DAS), emphasizing the agronomic importance of timely and sustained weed management under both sowing configurations.

### 3.5 Yield and yield-contributing traits

**Figure 4:**
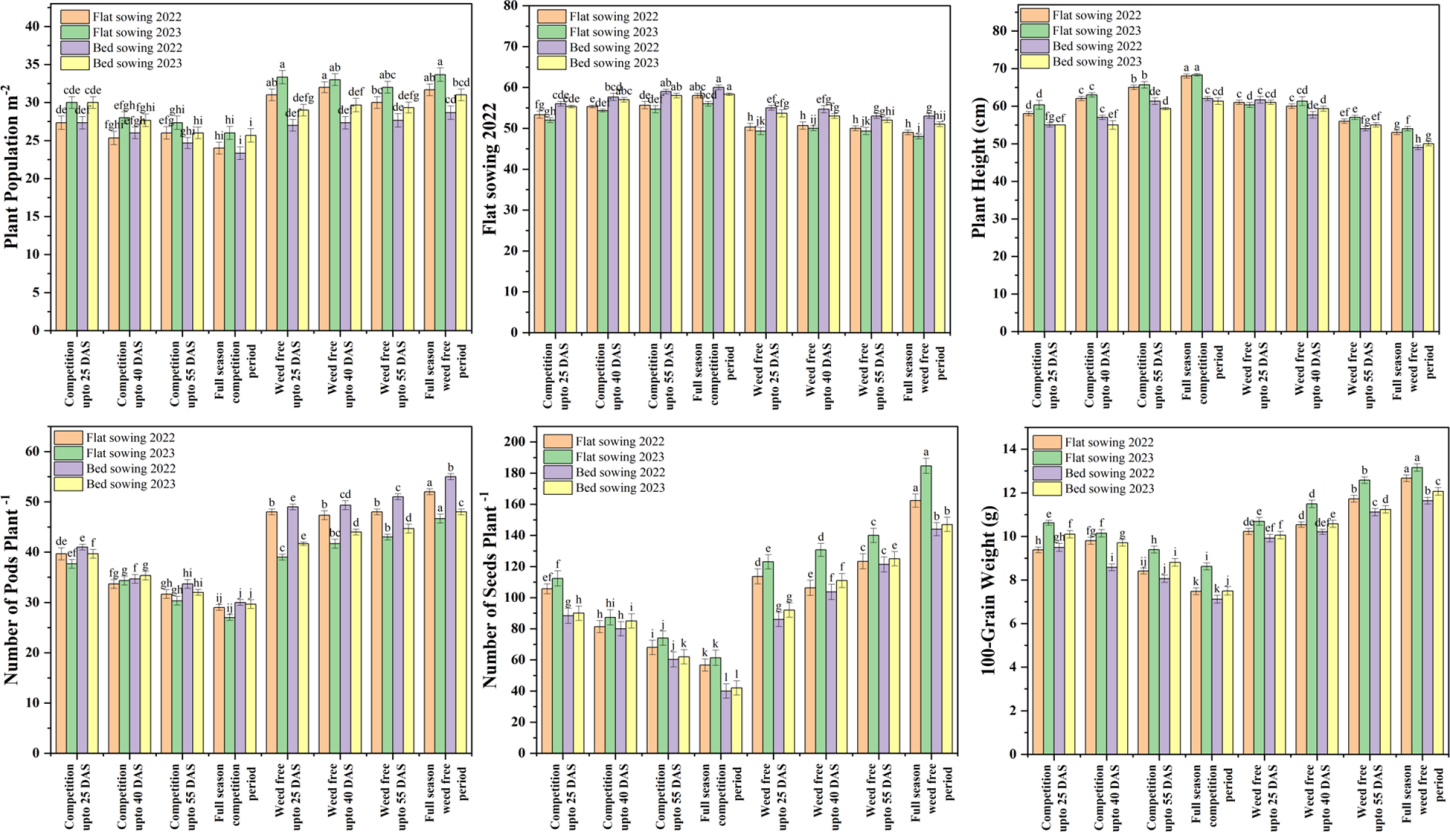
Soybean yield attributes response to sowing method and different period of weed interference in 2022 and 2023. Note: Error bars represent the standard deviation of three biological replicates. Bars (or points) sharing different lowercase letters are significantly different according to *post hoc* tests (*P<* 0.05).

Across both growing seasons, treatments with extended weed-free periods consistently promoted superior soybean growth and yield performance compared to limited weed control or continuous competition (**Figure 4**). In particular, flat sowing with “weed-free up to 25 DAS” and “full-season weed-free” treatments led to significantly higher plant populations (2022: 31.0 and 31.7 plants m^-2^; 2023: 33.3 and 33.7 plants m^-2^), more pods per plant (2022: 52 and 48; 2023: 55.0 and 49.0), greater seed numbers per plant (2022: 162.3 and 113.7; 2023: 184.7 and 123.0), and higher 100-grain weights (2022: 12.7 and 10.2 g; 2023: 13.2 and 10.7 g). These treatments also resulted in earlier flowering (2022: 49.0 and 50.3 days; 2023: 48.0 and 49.3 days). Conversely, bed sowing with restricted or no weed-free duration (“competition up to 40 DAS/55 DAS” and “full-season competition”) consistently resulted in lower plant populations (2022: 26.0, 24.7, and 23.3 plants m^-2^; 2023: 27.7, 26.0, and 25.7), fewer pods per plant (2022: 34.3, 30.3, and 27.0; 2023: 35.3, 32.0, and 29.7), reduced seeds per plant (2022: 80.0, 60.3, and 40.3; 2023: 85.0, 62.0, and 42.0), lighter 100-grain weights (2022: 8.6, 8.1, and 7.1 g; 2023: 9.7, 8.8, and 7.5 g), and delayed flowering (2022: 57.0, 59.0, and 60.0 days; 2023: 57.0, 58.0, and 58.0 days).

**Figure 5:**
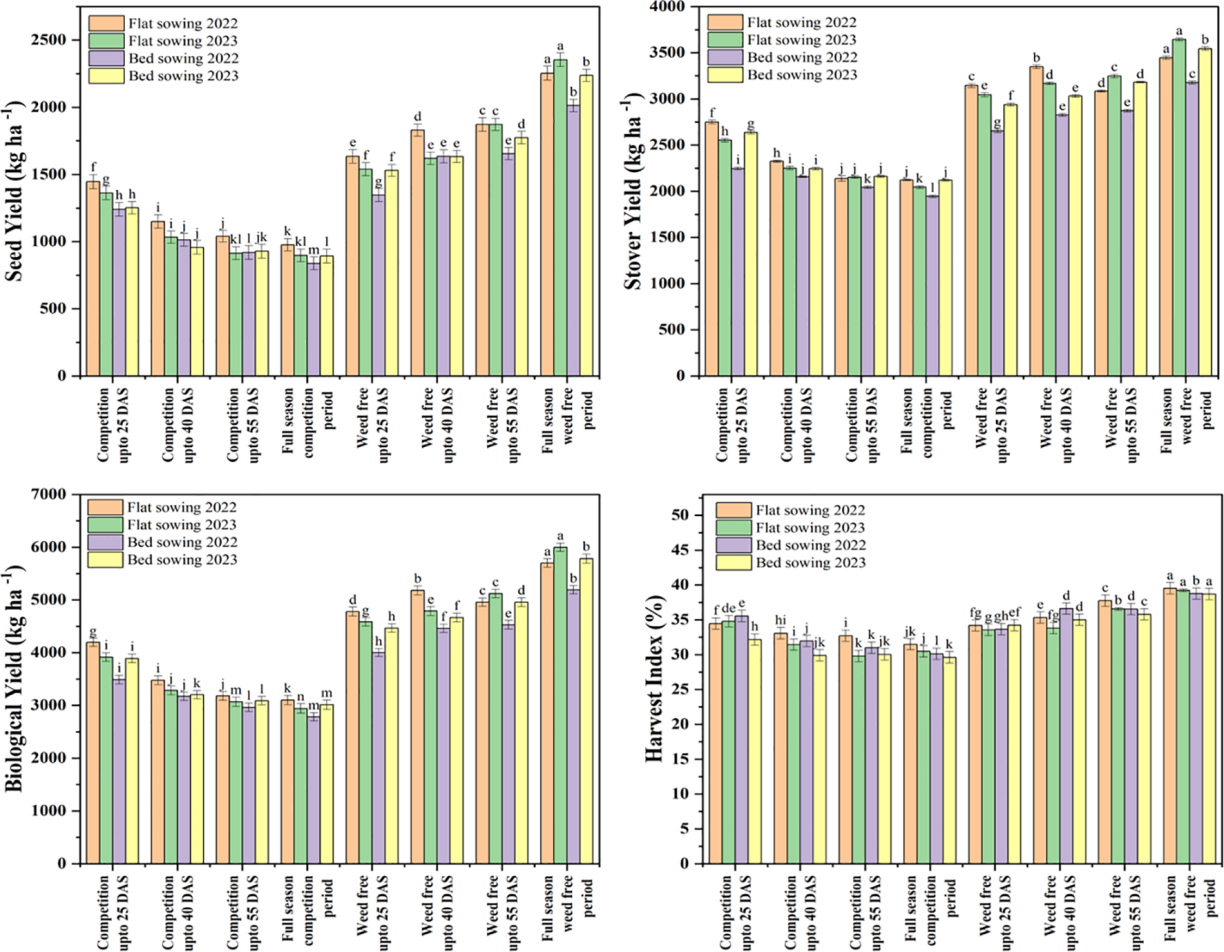
Soybean yield attributes response to sowing method and different period of weed interference in 2022 and 2023. Note: Error bars represent the standard deviation of three biological replicates. Bars (or points) sharing different lowercase letters are significantly different according to *post hoc* tests (*P<* 0.05).

Yield trends mirrored those of the growth parameters. Flat sowing with a 40 DAS weed-free period produced the highest seed yield (2022: 1,830.9 kg ha^-1^; 2023: 1,619.9 kg ha^-1^), stover yield (2022: 3,348.5 kg ha^-1^; 2023: 3,168.2 kg ha^-1^), biological yield (2022: 5,180.4 kg ha^-1^; 2023: 4,789.1 kg ha^-1^), and harvest index (2022: 35.34%; 2023: 33.83%) (**Figure 5**). In contrast, continuously weedy bed sowing plots (“competition up to 40 DAS/full season”) yielded the lowest values for these parameters. For example, in “competition up to 40 DAS,” seed yield was only 1,013.6 and 956.8 kg ha^-1^, stover yield 2,157.6 and 2,244.4 kg ha^-1^, biological yield 3,172.2 and 3,202.2 kg ha^-1^, and harvest index 31.95% and 29.88% in 2022 and 2023, respectively. The “full-season competition” treatment delivered the minimum yields across both years. Collectively, these results highlight that early and sustained weed control, especially between crop emergence and 40 DAS, substantially enhances soybean growth, accelerates phenological development, and maximizes yield potential, most notably under flat sowing methods.

### 3.6 Seed quality or biochemical traits

Analysis of quality parameters revealed that prolonged weed-free conditions, particularly up to 40 DAS, resulted in maximal soybean seed quality and oil yield across years (**Table 3**). Flat sowing with a 40 DAS weed-free period consistently produced the highest seed protein content (36.21% in 2022; 36.57% in 2023), oil content (19.38% in 2022; 19.78% in 2023), and oil yield (352.86 kg ha^-1^ in 2022; 318.42 kg ha^-1^ in 2023). These values are reported in comparison with all other treatments, including weed-free up to 25 DAS, 55 DAS, and full-season competition, as well as bed sowing under similar weed-free periods. These results were statistically at par with weed-free up to 55 DAS, underscoring the effectiveness of weed control sustained through early vegetative and branching stages. In contrast, continued weed competition dramatically reduced seed quality. The “full-season competition” treatment recorded the lowest protein content (32.34% in 2022; 33.84% in 2023), oil content (17.08% in 2022; 17.38% in 2023), and oil yield (171.07 kg ha^-1^ in 2022; 164.30 kg ha^-1^ in 2023). Collectively, these findings highlight that maintaining weed-free conditions through at least 40 DAS significantly enhances soybean seed protein, oil content, and oil yield. Conversely, extended weed competition substantially diminishes both yield and quality, reaffirming the agronomic and economic imperative for timely weed management in soybean cultivation.

**Table 3:** Soybean quality attributes response to sowing method and different period of weed interference in 2022 and 2023.

Treatments		Protein content (%)		Oil content (%)		Oil yield (kg ha^-1^)	
		2022	2023	2022	2023	2022	2023
Sowing method	Competition period						
Flat Sowing	Competition upto 25 DAS	34.23 ± 0.11^g^	35.29 ± 0.08^g^	18.10 ± 0.06^f^	18.19 ± 0.03^h^	259.90 ± 4.60^h^	245.79 ± 2.88^i^
	Competition upto 40 DAS	33.34 ± 0.11^i^	34.44 ± 0.11^h^	17.20 ± 0.05^g^	17.55 ± 0.08^j^	195.80 ± 2.01^k^	179.20 ± 2.19^k^
	Competition upto 55 DAS	32.30 ± 0.10^j^	33.62 ± 0.16^i^	17.12 ± 0.06^g^	17.15 ± 0.04^l^	176.03 ± 2.43^l^	154.77 ± 1.20^mn^
	Full season competition period	31.84 ± 0.09^k^	32.77 ± 0.07^j^	16.75 ± 0.10^h^	16.81 ± 0.06^m^	161.42 ± 0.20^m^	148.82 ± 1.07^no^
	Weed free upto 25 DAS	34.77 ± 0.13^f^	35.78 ± 0.12^f^	18.31 ± 0.09^e^	18.86 ± 0.06^f^	297.12 ± 0.94^g^	288.31 ± 2.44^g^
	Weed free upto 40 DAS	36.21 ± 0.10^d^	36.57 ± 0.16^e^	19.38 ± 0.08^c^	19.78 ± 0.07^d^	352.86 ± 4.34^d^	318.42 ± 0.41^e^
	Weed free upto 55 DAS	36.86 ± 0.09^c^	37.61 ± 0.17^cd^	20.03 ± 0.03^b^	20.57 ± 0.05^b^	372.98 ± 1.56^c^	383.26 ± 3.08^c^
	Full season weed free period	37.84 ± 0.11^a^	38.85 ± 0.09^a^	20.62 ± 0.07^a^	20.89 ± 0.05^a^	462.79 ± 6.27^a^	489.66 ± 3.09^a^
Bed Sowing	Competition upto 25 DAS	33.26 ± 0.15^i^	34.29 ± 0.08^h^	17.95 ± 0.04^f^	18.04 ± 0.03^i^	220.66 ± 3.87^j^	223.82 ± 2.50^j^
	Competition upto 40 DAS	32.34 ± 0.12^j^	33.84 ± 0.10^i^	17.08 ± 0.03^g^	17.38 ± 0.05^k^	171.07 ± 0.94^l^	164.30 ± 2.20^l^
	Competition upto 55 DAS	31.84 ± 0.10^k^	32.33 ± 0.11^k^	17.07 ± 0.07^g^	17.04 ± 0.03^l^	154.78 ± 0.87^m^	156.06 ± 1.79^m^
	Full season competition period	30.49 ± 0.15^l^	31.36 ± 0.12^l^	16.13 ± 0.06^i^	16.36 ± 0.07^n^	133.17 ± 1.51^n^	143.90 ± 1.52°
	Weed free upto 25 DAS	33.75 ± 0.13^h^	34.56 ± 0.13^h^	18.11 ± 0.07^ef^	18.55 ± 0.06^g^	241.93 ± 4.36^i^	281.85 ± 1.69^h^
	Weed free upto 40 DAS	35.59 ± 0.09^e^	37.41 ± 0.15^d^	19.15 ± 0.08^d^	19.23 ± 0.06^e^	311.06 ± 3.26^f^	312.19 ± 2.99^f^
	Weed free upto 55 DAS	36.40 ± 0.15^d^	37.90 ± 0.06^bc^	19.96 ± 0.04^b^	20.18 ± 0.04^c^	328.38 ± 2.98^e^	356.04 ± 2.00^d^
	Full season weed free period	37.29 ± 0.07^b^	38.23 ± 0.12^b^	20.13 ± 0.06^b^	20.55 ± 0.07^b^	403.15 ± 2.26^b^	457.78 ± 2.38^b^
	MS for sowing methods (S)	7.7715**	4.7125**	0.6953**	1.1544**	18570.5**	2364.7**
	MS for competition period (CP)	30.7244**	31.5928**	12.3816**	13.9843**	60938.0**	79726.1**
	MS for S × CP	0.1609**	0.9737**	0.0633**	0.0359**	291.6**	212.6**
	LSD At 5%	0.3421	0.3658	0.2157	0.1422	8.7763	5.8682

Error bars represent the standard deviation of three biological replicates. Bars (or points) sharing different lowercase letters are significantly different according to *post hoc* tests (*P<* 0.05).

### 3.7 Pearson correlation matrices

**Figure 6:**
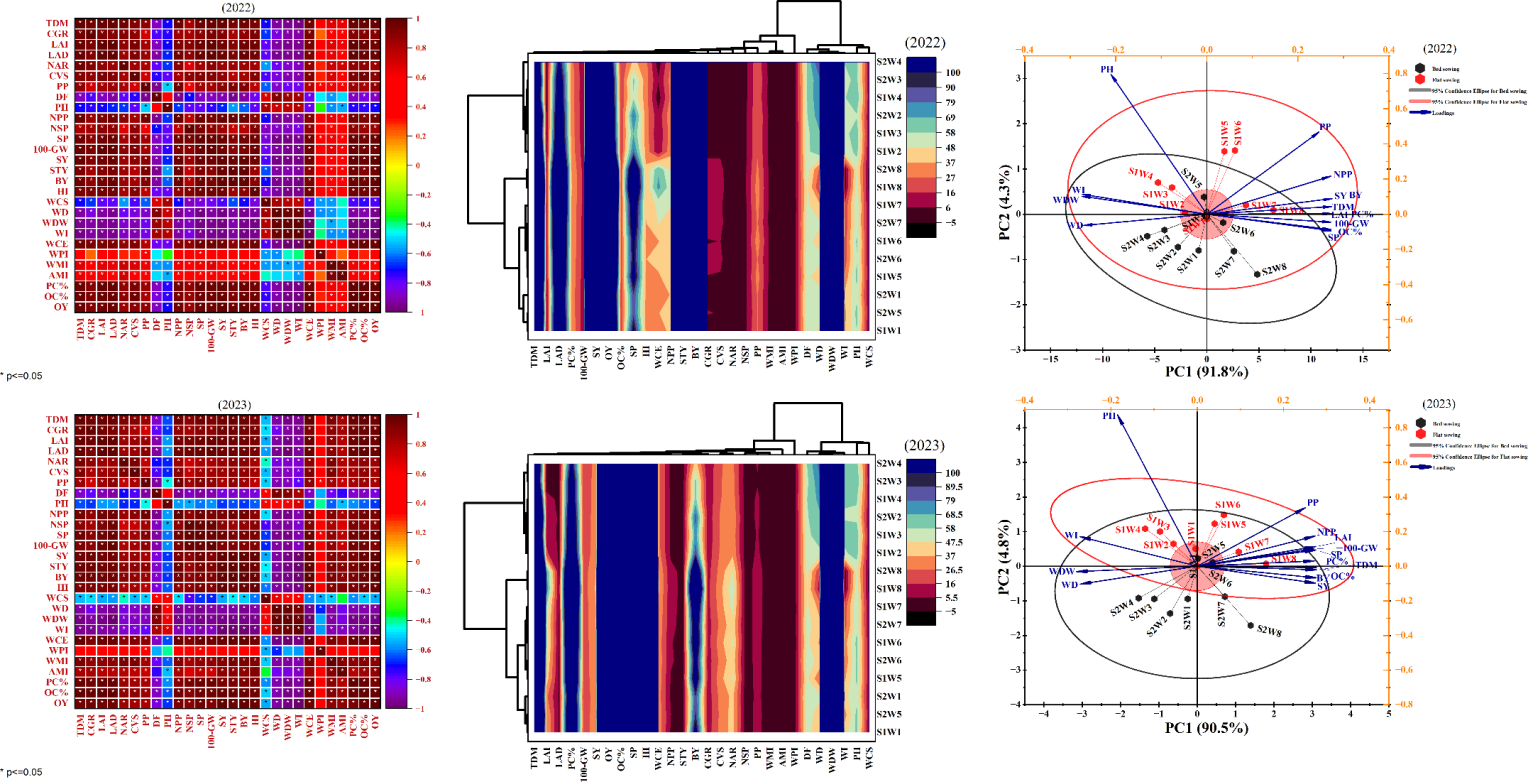
Pearson correlation, Clustered heat map and Principal component analysis (PCA) of weeds attributes and soybean growth, yield, and quality parameters under flat and bed sowing methods during 2022 and 2023. TDM (total dry matter), CGR (crop growth rate), LAD (leaf area duration), NAR (net assimilation rate), CVS (crop vigor score), PP (plant population) DF (days to flowering), PH (plant height), NPP (number of pods per plant), NSP (number of seeds per pod), SP (number of seeds per plant), 100-GW (100 grains weight) SY (seed yield) STY (stover yield) BY (biological yield) HI (harvest index) WCS (weed cover score) WD (weeds density) WDW (weeds dry weight), WI (weed index), WCE (weed control efficiency), WPI (weed persistence index), WMI (weed management index), AMI (agronomic management index), PC (protein content), OC (oil content), OY (oil yield). Whereas, S1 (Flat Sowing) and S2 (Bed Sowing), W1 (Competition up to 25 DAS), W2 (Competition up to 40 DAS), W3 (Competition up to 55 DAS), W4 (Full season competition period), W5 (Weed-free up to 25 DAS), W6 (Weed-free up to 40 DAS), W7 (Weed-free up to 55 DAS) and W8 (Full season weed-free period).

Pearson correlation analyses for 2022 and 2023 revealed clear and consistent relationships between soybean growth, yield, and quality parameters and various weed attributes, with the results visualized by color-coded matrices (**Figure 6**: red = positive correlation, blue = negative correlation). Across both years, TDM was strongly and positively correlated with key growth indices, including CGR, LAD, and NAR, indicating that greater biomass accumulation was consistently associated with improved physiological performance. Plant height and pod number per plant also showed significant positive correlations with these growth variables. In contrast, WD and WDW exhibited significant negative correlations with most morphological, yield, and quality characteristics, confirming that increased weed pressure markedly reduces soybean productivity. WCE was negatively correlated with weed attributes such as WCS and WPI in both years, underscoring the effectiveness of timely and sustained weed management. While the overall pattern of correlations remained stable from year to year, some variation was observed, notably in the relationships between quality traits, such as protein content (PC%) and oil content (OC%), and other variables. These shifts may reflect annual environmental differences or subtle changes in management practices. Taken together, the correlation matrices indicate that effective weed suppression enhances soybean growth, yield, and quality, while weed pressure consistently undermines crop performance. The persistence of these relationships across seasons highlights the central importance of integrated weed management for sustainable soybean production.

### 3.8 Clustered heat maps

The clustered heat maps for 2022 and 2023 provided an integrated visualization of the relationships between weed attributes and key soybean growth, yield, and quality parameters across sowing methods and weed competition regimes (**Figure 6**). Clear clustering patterns revealed that extended weed competition, especially under full-season competition periods (W4), consistently led to elevated WCS and WD, irrespective of sowing method. These clusters highlight the suppressive effect of prolonged weed pressure on crop performance. Conversely, weed-free treatments, notably W7 (weed-free up to 55 DAS) and W8 (full-season weed-free), formed distinct clusters characterized by markedly reduced weed pressure and enhanced crop performance. Under these conditions, yield components such as total dry matter, crop growth rate, and seed yield reached their highest levels, particularly with flat sowing (S1), which consistently grouped with superior growth and productivity metrics. Quality parameters, including protein content and oil yield, were similarly highest in weed-free treatments, particularly under flat sowing, indicating that effective weed control is integral not only for yield but also for seed quality. Traits such as leaf area duration, net assimilation rate, and crop vigor score also clustered with the weed-free groups, further underscoring the holistic benefits of timely weed management. Notably, management indices (AMI and WMI) were substantially greater in weed-free treatments, affirming the agronomic and economic advantages of early and sustained weed removal. Patterns and clusters were broadly consistent across both years, though some variation in intensity reflected annual climatic and management differences. Overall, the clustered heat maps demonstrate that maintaining weed-free conditions, especially with flat sowing, optimizes soybean growth, yield, and quality, whereas delayed or inadequate weed control significantly diminishes crop performance.

### 3.9 Principal component analysis

Principal component analysis (PCA) was conducted to distil the relationships among soybean growth, yield, quality attributes, and weed pressure across sowing methods and weed management regimes in 2022 and 2023 (**Figure 6**). In both years, the first principal component (PC1) accounted for the vast majority of variance (91.8% in 2022; 90.5% in 2023), while PC2 explained 4.3% and 4.8%, respectively. The PCA biplots revealed that significant productivity-related variables, including TDM, CGR, LAD, SY, and BY, clustered strongly along PC1, reflecting their central role in driving overall soybean performance. In sharp contrast, weed attributes such as WCS, WD, and WDW were negatively associated with PC1, indicating that increased weed pressure is consistently linked to suppressed crop productivity. Treatment positioning in the biplots further clarified these dynamics. Flat sowing (S1) under extended weed-free conditions was distinctly separated from high-weed-pressure treatments (notably bed sowing with full-season competition), occupying regions of the PCA space associated with enhanced growth and yield metrics. This separation was statistically supported by non-overlapping 95% confidence ellipses for flat and bed sowing, reinforcing the robustness of these distinctions across both study years. Overall, the PCA results demonstrate that adopting flat sowing in combination with practical and timely weed management maximizes soybean growth, yield, and quality by mitigating the adverse effects of weed competition. The consistency in variable clustering and treatment separation across both years highlights the reliability and practical significance of these findings for sustainable soybean production.

### 3.10 Linear regression

**Figure 7:**
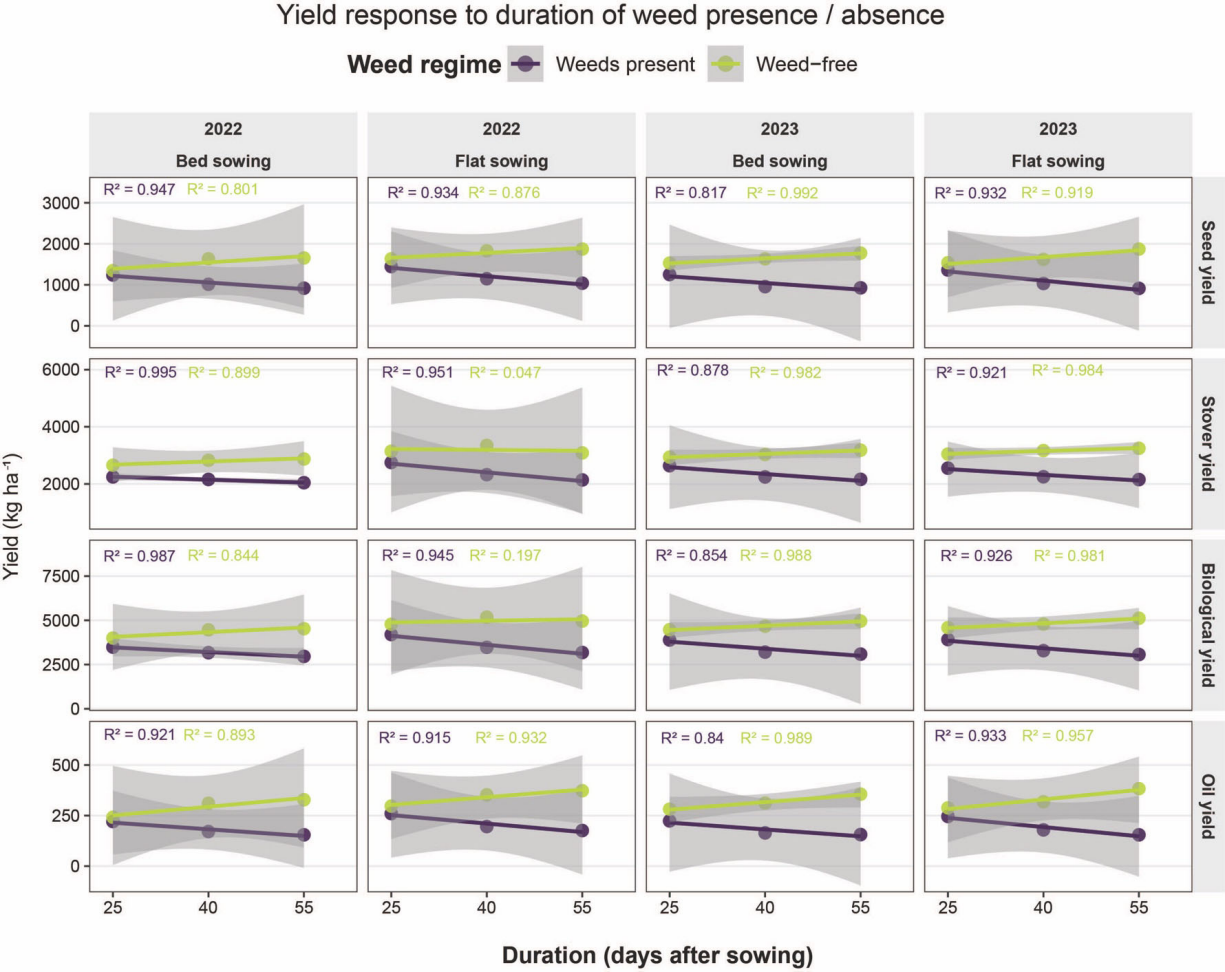
Linear regression of seed, stover, biological and oil yields in relation to the duration of weed presence and weed-free periods (25, 40 and 55 days after sowing) under bed and flat sowing during 2022 and 2023. R² values indicate the strength of the relationships.

Regardless of sowing method and experimental year, all yield components (seed, stover, biological and oil yield) were consistently higher under weed-free conditions compared with weedy treatments. Linear regression analysis indicated a positive trend in yield with increasing duration of weed-free periods, whereas a clear negative trend was observed under weed competition. Yield declined progressively as the duration of weed interference increased from 25 to 55 days after sowing, reflecting the cumulative adverse effects of prolonged weed-crop competition on crop growth and productivity. The high R² values in most cases further confirm the strong linear relationship between weed interference duration and yield reduction, as well as between extended weed-free periods and yield improvement (**Figure 7**).

## 4 Discussion

### 4.1 Weed flora dynamics and seasonal influence

During the two-year study, a total of thirteen weed species were documented, comprising seven broadleaf species, three grasses, and three sedges, reflecting the diversity typically favored by highly disturbed agroecosystems. These weed species, known for their competitive traits such as rapid growth, high reproductive potential, and adaptive morphology, can strongly influence crop performance depending on their density and emergence timing. Environmental factors, particularly rainfall patterns, interact with these weed traits to modulate their competitiveness. For instance, abundant and well-distributed rainfall can enhance early-season weed establishment, increasing resource competition with soybeans. Conversely, less favorable conditions may limit weed growth, indirectly benefiting crop performance (Lososová and Cimalová, 2009). Overall, understanding the ecological traits of prevalent weeds and their interaction with seasonal weather provides critical context for designing timely and effective weed management strategies in soybean cultivation. This contextual insight goes beyond simply reporting infestation levels, offering guidance on how weed pressure may vary under different agroecological conditions (Daramola, 2020). Datta et al. (2017) reported that intensively managed arable systems are typically dominated by a limited number of highly competitive annual broadleaf and grass weed species, particularly under frequent soil disturbance and nutrient-rich conditions. In their Central European surveys, weed communities were strongly shaped by rainfall distribution and temperature regimes, with wetter seasons favoring early-emerging broadleaf species. Similarly, Monah et al. (2025) observed that seasonal rainfall variability significantly altered weed species composition and density in soybean-based systems, with higher rainfall years supporting greater weed biomass and prolonged emergence periods.

### 4.2 Impact of sowing methods and weed-free periods on weed suppression

Our findings demonstrate that flat sowing, combined with a weed-free period of up to 40 DAS, was most effective in minimizing weed cover score, density, and dry weight, highlighting the critical importance of early and consistent weed management in soybean production. This effectiveness can be attributed to several mechanistic factors, including faster canopy closure in flat sowing due to narrower row spacing, which allows soybean seedlings to occupy available space rapidly and outcompete emerging weeds for light, water, and nutrients (Hazra et al., 2009). By suppressing weeds early, the soybean crop gains a significant competitive advantage during its most vulnerable early growth stages (Štefanić et al., 2022). Simultaneously, the extensive root system of well-established soybean plants intensifies competition for soil moisture and nutrients, further limiting weed proliferation (Rosset and Gulden, 2019).

In contrast, bed sowing combined with delayed or insufficient weed control was associated with higher weed cover and density, largely because wider row spacing in beds slows canopy closure, leaving more open space for weeds to establish. Incremental weed-pressure treatments in our study clearly demonstrated that even short periods of weed presence (25 or 40 DAS) allowed weeds to gain a competitive foothold compared with the no-weed and full-season competition checks. Overall, the integration of flat sowing and timely weed removal triggers a cascade of competitive and ecological mechanisms, spanning early resource preemption, rapid canopy development, and effective physical exclusion, that together ensure robust, season-long weed suppression in soybean. Singh et al. (2023) demonstrated that narrower row spacing in soybean significantly reduced weed density and biomass by accelerating canopy closure, which limited light availability at the soil surface during early crop growth. Štefanić et al. (2022) similarly reported that maintaining soybean fields weed-free during the first 4–6 weeks after emergence resulted in substantially lower weed pressure and improved crop competitiveness, confirming the importance of early-season weed control. Jha and Soni (2013) further showed that soybean root systems expand more rapidly under reduced weed interference, enhancing belowground competition for water and nutrients and suppressing late-emerging weeds.

### 4.3 Soybean growth and physiological response to weed competition

Elevated values of key growth parameters, such as crop vigor score, LAI, and CGR, were consistently observed under flat sowing with a weed-free period extending to 40 DAS across both study years. The 40 DAS weed-free treatment showed superior growth compared with the 25 DAS weed-free, 55 DAS weed-free, and full-season competition treatments, indicating that early weed control in the vegetative stage is critical for maximizing soybean canopy development and growth potential. In competitive conditions, weeds aggressively exploit soil moisture and nutrients near the surface, casting shade over the developing soybean canopy and directly suppressing root expansion, thereby impeding efficient nutrient uptake (Horvath et al., 2015). At a physiological and molecular level, soybean plants subjected to weed competition initiate stress responses, including the upregulation of transcription factors such as *MYB* and *WRKY*, which are well known for orchestrating plant defense at the expense of vegetative growth (Liu et al., 2003). Moreover, weed-induced stress enhances oxidative stress responses and triggers jasmonic acid signaling pathways, as reflected by the increased activity of genes like *PIF3*, which divert plant energy from growth towards defense (Nan et al., 2014).

Mechanistically, progressive improvements in LAI and CGR were observed as the weed-free period increased from 25 DAS to 40 DAS. However, extending the weed-free duration beyond 40 DAS (to 55 DAS or full-season weed-free) did not yield statistically significant additional benefits, indicating an optimal window for weed management. This benefit is underpinned by the plants’ ability to invest photosynthetic resources toward expanding the canopy in the absence of competitive stress, leading to improved overall photosynthetic efficiency and resource assimilation (Smith et al., 2023). Supporting this, the expression of key regulatory genes, such as *GmFT2a*, which modulates leaf area expansion, remains less constrained when weeds are excluded, resulting in more vigorous vegetative development (Jabran et al., 2015). CGR was similarly enhanced under prolonged weed-free conditions, driven by unimpeded access to growth resources and the absence of allelopathic constraints from neighboring weeds (Chen et al., 2022). This is especially noteworthy, given that allelochemicals, such as caffeic acid from weed species like *Artemisia argyi* L., have been shown to suppress crop growth by downregulating genes involved in gibberellin and phytoalexin biosynthesis, as well as disrupting stress-responsive MAP kinase pathways (Le et al., 2011). Under reduced competition, soybeans exhibit higher expression of growth hormone-related genes (e.g., those involved in gibberellin biosynthesis), further promoting rapid biomass accumulation and growth (Cadavid et al., 2023). Collectively, these findings demonstrate that the progressive differences in growth response across treatments underscore the importance of early weed control, with the 40 DAS weed-free period representing the most effective strategy for maximizing vegetative development, leaf expansion, and crop vigor. These effects are driven not only by improved resource capture above and below ground but also by favorable shifts in plant molecular signaling and hormonal status, highlighting the integrated physiological and genetic advantages of early and sustained weed management in soybean cultivation. Omarov et al. (2019) reported that early-season weed competition significantly reduced soybean LAI and biomass accumulation by limiting light interception and nutrient uptake, particularly during vegetative stages. At the physiological level, Caldas et al. (2023) showed that weed-induced stress activated MYB and WRKY transcription factors in soybean, leading to growth suppression and altered carbon allocation. Bradley et al. (2002) further demonstrated that competitive stress enhanced jasmonic acid signaling and oxidative stress responses, diverting metabolic resources away from vegetative growth. Gawęda and Kopcewicz (2023) found that extending the weed-free period beyond the early vegetative stage improved LAI and CGR, but gains diminished once canopy closure was achieved, supporting our observation that weed-free conditions beyond 40 DAS provided no additional growth advantage.

### 4.4 Yield and quality enhancement under optimal weed management

Our results demonstrate that maintaining a weed-free period up to 40 DAS, particularly under flat sowing, significantly improved soybean yield and all major yield components, including number of seeds per pod, pods per plant, grains per plant, and 100-grain weight, relative to season-long weed competition or shorter weed-free durations (Peer et al., 2012). This yield improvement is primarily attributed to reduced early-season competition, which enabled soybean plants to utilize essential resources—such as light, nutrients, and water—more efficiently (Keerthana et al., 2021). Moreover, recent evidence indicates that even plant-to-plant spatial uniformity within soybean stands plays a critical role in determining yield potential during the early growth period. Pereyra et al. (2022) demonstrated that non-uniform spacing among soybean plants can significantly reduce yields, particularly under low and medium-yielding environments and reduced plant densities, underscoring the sensitivity of soybean to early-season competitive pressures. These findings reinforce the importance of minimizing all forms of early-season interference—whether from weeds or uneven crop establishment—to ensure optimal resource capture. The physiological mechanisms underpinning these outcomes include enhanced photosynthesis, supported by greater leaf area development and more effective light interception, as well as increased nutrient uptake resulting from unimpeded root growth (Sepat et al., 2017). Additionally, the early establishment of a vigorous soybean canopy under weed-free conditions also physically suppressed weed emergence (smothering effect), amplifying the benefits of direct weed control (Zhou et al., 2008).

The observed improvements in growth and stress tolerance under weed-free conditions are consistent with previous studies showing that reduced competition can influence the expression of growth- and stress-related genes, such as GmWRKY13, GmWRKY21, GmWRKY54, and GmFT2a, which support lateral root development, stress resilience, and efficient resource allocation for seed development (Wang et al., 2008; Moriles et al., 2012; Benemann et al., 2020). In contrast, prolonged weed competition has been associated with downregulation of photosynthesis-related genes (e.g., GmPHYA) and activation of stress-response pathways, which may divert resources from productive growth to defense, limiting biomass and seed yield (Spaeth et al., 1984; Nimu et al., 2020). Soybean quality parameters, including seed protein content, oil content, and oil yield, were highest in extended weed-free plots, particularly with flat sowing up to 40 DAS. These improvements likely result from enhanced nutrient (especially nitrogen) uptake and increased carbon assimilation, both of which can be constrained under weed-infested conditions due to competition and allelopathic effects (Gadakh et al., 2020). Literature further suggests that genes involved in protein (e.g., GmNAC2, GmDOF4) and oil biosynthesis (e.g., GmFAD2, GmOLE1) are more active under low-competition scenarios, contributing to improved seed quality (Millar et al., 2007).

Resource competition in the presence of weeds may also be accompanied by allelopathic interactions, as some dominant species are reported in the literature to release phenolic acids, terpenoids, and other allelochemicals that can suppress soybean germination and early seedling growth (Apon and Nongmaithem, 2022). Although allelochemicals were not measured in the present study, reducing weed pressure during the critical window (25–40 DAS) likely minimized both competitive stress and any potential allelopathic interference (Zhang et al., 2014). The competitive advantage observed with flat sowing, compared to bed sowing, is likely due to denser initial crop stands, faster canopy closure, and strategic irrigation timing, all of which compound weed suppression and maximize resource use efficiency (Daugovish et al., 2003; Arias et al., 2022; Gawęda et al., 2024). Taken together, these findings strongly support the adoption of flat sowing combined with timely weed management during the early vegetative phase as a best practice for non-GMO soybean production. Such an integrated strategy not only maximizes soybean yield and quality but also enhances resource-use efficiency, providing agronomic resilience in variable environmental conditions. Morsy and Tantawy (2018) reported yield losses of up to 35–45% in soybean when weeds were allowed to compete beyond the early vegetative stage, emphasizing the importance of maintaining a weed-free period during the first 30–40 DAS. Similarly, Rowland et al. (2023) showed that early weed removal significantly increased pods per plant and 100-seed weight by improving early-season resource availability. El-Metwally et al. (2022) demonstrated that even minor spatial interference during early growth stages reduced soybean yield potential, reinforcing the sensitivity of soybean to early competition observed in the present study. Moreover, studies further linked improved yield under weed-free conditions to enhanced photosynthetic efficiency and nutrient uptake (Horvath et al., 2023), while Barla et al. (2022) documented a strong smothering effect of early canopy closure on late-emerging weeds, contributing indirectly to yield stability.

### 4.5 Strategic implications for sustainable agriculture and future research

While this study provides clear evidence that flat sowing, combined with an early weed-free period (particularly up to 40 DAS), offers substantial advantages for soybean growth, yield, and quality, several limitations should be acknowledged. First, the experiments were conducted over two consecutive seasons at a single research station under specific climatic and edaphic conditions. As such, the findings may not be fully generalizable to other geographic regions, soil types, or under contrasting weather patterns, including years with more erratic rainfall or temperature extremes. Manual weed control was the primary intervention in this study, which may limit the direct applicability of the results for large-scale or resource-constrained farmers where labor shortages or costs are a practical concern. The weed community documented here may also differ from that present in other cropping systems or under changed climate and management regimes, and interactions with new or evolving weed populations cannot be discounted.

Moving forward, future research should prioritize multi-location and multi-year trials to assess the robustness and transferability of these weed management strategies across varying agroecological environments. There is a need to integrate a broader range of weed management technologies, including mechanical, cultural, biological, and, where suitable, environmentally responsible chemical methods, to develop holistic, scalable, and cost-effective weed control packages. Economic evaluations should be embedded to assess not only yield benefits but also labor requirements, input costs, and overall profitability. More nuanced research could further explore the physiological, molecular, and ecological mechanisms underlying crop–weed competition, with a focus on understanding the interaction between critical weed-free periods and changing weed flora under future climate scenarios.

Emerging technologies, such as precision agriculture tools, remote sensing, and decision-support models, offer promising avenues for optimizing the timing and intensity of weed management, thereby minimizing inputs while maximizing returns. Biological control agents, allelopathy-based strategies, and competitive cultivars may also play an increasing role as components of integrated weed management. Finally, participatory research with farmers will be crucial to ensure that recommended practices are both agronomically effective and suitable for adoption at scale. In summary, while early and sustained weed control, especially in flat-sown systems, represents a robust pathway to maximize non-GMO soybean performance in the context studied here, broader adaptive research and innovation remain essential to meet the evolving needs and challenges of sustainable soybean production.

## 5 Conclusions

This study demonstrates that both the timing and method of weed management are decisive in shaping soybean yield and seed quality. Among all tested practices, flat sowing with a weed-free period extended up to 40 DAS resulted in the highest plant population, pod development, 100-grain weight, grain and stover yield, and enhanced seed protein and oil content. By maintaining the crop free of weed competition between 25 and 55 DAS, the most sensitive period for soybean resource use, yield losses were minimized. Data show that uncontrolled weed growth throughout the season can reduce yield by nearly 60%. Conversely, weed removal after 40 DAS or before 25 DAS conferred no significant benefit, indicating that resources for weed management should be targeted to this critical window. These findings highlight the practical importance of flat sowing and timely, sustained weed control in maximizing soybean productivity under the studied agroecological conditions. The results provide clear guidance for growers: prioritize early weed-free periods, particularly through flat sowing, to safeguard and optimize yield and grain quality. Further research is warranted to test these strategies under broader climatic and soil conditions, and to develop integrated, site-specific weed management approaches that combine mechanical, chemical (where appropriate), and ecological practices. Exploring the long-term effects of such management on soil health, weed dynamics, and economic returns will be essential for sustainable soybean production in diverse farming systems.

## Data Availability

The original contributions presented in the study are included in the article/**Supplementary Material**. Further inquiries can be directed to the corresponding authors.

## Supplementary material

Supplementary Table 1 Weed species and their level of infestation during the period of crop growth in 2022 and 2023.*** Highly infested (60–90); ** Moderately infest (30–59%); * Low infestation (1–29%); ^-^ not noticeable.
