# Supplementary material for: Enhancing soybean (Glycine max L.) yield and quality through optimized weed-free periods and sowing techniques
Source: Front Plant Sci. 2026 Feb 16;17:1700878. doi: 10.3389/fpls.2026.1700878 (PMC12951636; doi:10.3389/fpls.2026.1700878)
Supplement: Supplementary Table 1 — Weed species and their level of infestation during the period of crop growth in 2022 and 2023.*** Highly infested (60–90); ** Moderately infest (30–59%); * Low infestation (1–29%); - not noticeable. [file Table1.docx]

Supplementary Material

**
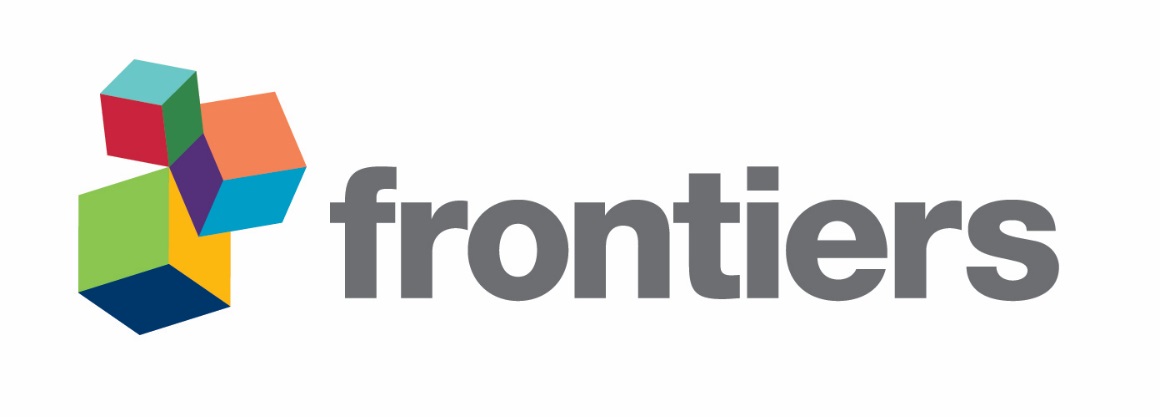
**

**Supplementary Table 1:** ***Weed species and their level of infestation during the period of crop growth in 2022 and 2023.***

| **Weed species** | **Plant family** | **Level of infestation** | |
| --- | --- | --- | --- |
|  |  | **2022** | **2023** |
| **Broad leaf weeds** | | | |
| Trianthema portulacastrum (Linn). | Aizoaceae | *** | *** |
| *Convolvulus arvensis* (Linn). | Convolvulaceae | *** | *** |
| *Tribulus terrestris* L. | Zygophyllaceae | *** | ** |
| *Xanthium strumarium* L. | Compositae | ** | ** |
| *Euphorbia granulate* L. | Euphorbiaceae | ** | * |
| *Euphorbia hirta* L. | Euphorbiaceae | - | * |
| *Parthenium hysterophorus* L. | Asteraceae | * | * |
| **Grasses** | | | |
| *Cynodon dactylon* (L) Gaertn | Poaceae | *** | *** |
| *Paspalum distichum* (L). | Poaceae | - | * |
| Phalaris minor Retz. | Poaceae | * | * |
| **Sedges** | | | |
| *Cyperus rotundus* | Cyperaceae | *** | *** |
| *Cyperus esculentus* | Cyperaceae | * | - |
| *Cyperus difformis* | Cyperaceae | - | * |

******* Highly infested (60-90); ****** Moderately infest (30-59%); * Low infestation (1-29%); **^-^** not noticeable.
